# Incident heart failure and myocardial infarction in sodium‐glucose cotransporter‐2 vs. dipeptidyl peptidase‐4 inhibitor users

**DOI:** 10.1002/ehf2.13830

**Published:** 2022-02-07

**Authors:** Jiandong Zhou, Sharen Lee, Keith Sai Kit Leung, Abraham Ka Chung Wai, Tong Liu, Ying Liu, Dong Chang, Wing Tak Wong, Ian Chi Kei Wong, Bernard Man Yung Cheung, Qingpeng Zhang, Gary Tse

**Affiliations:** ^1^ Nuffield Department of Medicine University of Oxford Oxford UK; ^2^ Diabetes Research Unit Cardiovascular Analytics Group Hong Kong China; ^3^ Emergency Medicine Unit, Faculty of Medicine The University of Hong Kong Hong Kong China; ^4^ Tianjin Key Laboratory of Ionic‐Molecular Function of Cardiovascular Disease, Department of Cardiology, Tianjin Institute of Cardiology Second Hospital of Tianjin Medical University Tianjin China; ^5^ Department of Cardiology The First Affiliated Hospital of Dalian Medical University Dalian China; ^6^ Xiamen Cardiovascular Hospital Xiamen University Xiamen China; ^7^ School of Life Sciences, State Key Laboratory of Agrobiotechnology (CUHK) The Chinese University of Hong Kong Hong Kong China; ^8^ Department of Pharmacology and Pharmacy University of Hong Kong Hong Kong China; ^9^ Division of Clinical Pharmacology and Therapeutics, Department of Medicine The University of Hong Kong Hong Kong China; ^10^ School of Data Science City University of Hong Kong Hong Kong China; ^11^ Kent and Medway Medical School Canterbury Kent UK

**Keywords:** Sodium‐glucose co‐transporter, Heart failure, Myocardial infarction, Diabetes mellitus

## Abstract

**Aims:**

This study aimed to compare the rates of major cardiovascular adverse events in sodium‐glucose cotransporter‐2 inhibitors (SGLT2I) and dipeptidyl peptidase‐4 inhibitors (DPP4I) users in a Chinese population. SGLT2I and DPP4I are increasingly prescribed for type 2 diabetes mellitus patients. However, few population‐based studies are comparing their effects on incident heart failure or myocardial infarction.

**Methods and results:**

This was a population‐based retrospective cohort study using the electronic health record database in Hong Kong, including type 2 diabetes mellitus patients receiving either SGLT2I or DPP4I from 1 January 2015 to 31 December 2020. Propensity score matching was performed in a 1:1 ratio based on demographics, past comorbidities, and non‐SGLT2I/DPP4I medications with nearest neighbour matching (caliper = 0.1). Univariable and multivariable Cox models were used to identify significant predictors for new‐onset heart failure, new‐onset myocardial infarction, cardiovascular mortality, and all‐cause mortality. Sensitivity analyses with competing risk models and multiple propensity score matching approaches were conducted. A total of 41 994 patients (58.89% males, median admission age at 58 years old, interquartile range [IQR]: 51.2–65.3) were included with a median follow‐up of 5.6 years (IQR: 5.32–5.82). In the matched cohort, SGLT2I use was significantly associated with lower risks of new‐onset heart failure (hazard ratio [HR]: 0.73, 95% confidence interval [CI]: [0.66, 0.81], *P* < 0.0001), myocardial infarction (HR: 0.81, 95% CI: [0.73, 0.90], *P* < 0.0001), cardiovascular mortality (HR: 0.67, 95% CI: [0.53, 0.84], *P* < 0.001), and all‐cause mortality (HR: 0.26, 95% CI: [0.24, 0.29], *P* < 0.0001) after adjusting for significant demographics, past comorbidities, and non‐SGLT2I/DPP4I medications.

**Conclusions:**

SGLT2 inhibitors are protective against adverse cardiovascular events including new‐onset heart failure, myocardial infarction, cardiovascular mortality, and all‐cause mortality. The prescription of SGLT2I is preferred when taken into consideration individual cardiovascular and metabolic risk profiles in addition to drug–drug interactions.

## Introduction

Diabetes mellitus is an increasingly prevalent metabolic disease, currently affecting more than 400 million people, and the patient population is projected to increase up to 642 million by 2040.^1^ Given the ever‐increasing disease burden, new classes of antidiabetic agents have been introduced into the market over the past decade. The use of two novel classes of antidiabetic agents—sodium‐glucose cotransporter‐2 inhibitors (SGLT2I) and dipeptidyl peptidase‐4 inhibitors (DPP4I)—has increased significantly.^2^, ^3^ Besides their favourable side effect profile, studies have reported beneficial effects on metabolic risk from these two classes of drugs.^4^ Based on findings from large‐scale clinical trials, the cardiovascular mortality‐lowering effects of SGLT2I are mostly attributed to its protection against heart failure (HF).^5^, ^6^, ^7^, ^8^ On the other hand, the cardiovascular effect of DPP4I appears to be more controversial. Whilst there were reports of DPP4I users having lower cardiovascular risks than non‐users, there are also studies reporting an increased risk of HF in saxagliptin users.^9^, ^10^

Whilst small‐scale trials are comparing the metabolic effects or specific disease outcomes of SGLT2I and DPP4I, there is a lack of large‐scale population studies to evaluate the difference in the presentation of major cardiovascular adverse events between the use of the two drug classes.^11^, ^12^, ^13^ Recently, Zheng *et al*. have demonstrated lower mortality in SGLT2I users in comparison with DPP4I users in a network meta‐analysis.^14^ However, ultimately, the study is limited by the indirect comparison of the SGLT2I and DPP4I users. Other studies have reported on outcomes such as weight loss, improvement in the liver or renal function,^15^ and reduction in atrial fibrillation incidence.^16^ Another study recently investigated cardiovascular outcomes such as HF and myocardial infarction (MI), but only in Japanese, Korean, and European cohorts.^17^ Therefore, the aim of the present study is to compare the occurrence of major cardiovascular adverse events in SGLT2I and DPP4I users to evaluate their cardiovascular protective effects in a Chinese population.

## Methods

### Study design and population

This study was approved by the Institutional Review Board of the University of Hong Kong/Hospital Authority Hong Kong West Cluster and from The Joint Chinese University of Hong Kong–New Territories East Cluster Clinical Research Ethics Committee. It included type 2 diabetes mellitus patients with SGLT2I or DPP4I prescriptions from 1 January 2015 to 31 December 2020. Patients who received both DPP4I and SGLT2I, in addition to patients who discontinued the medication during the study, were excluded. The exclusion criteria for the HF study cohort were as follows: patients with prior HF diagnosis or with the use of medications for HF (e.g. diuretics for HF and beta‐blockers for HF). For the MI study cohort, patients with prior old MI or MI diagnosis were excluded. The patients were identified from the Clinical Data Analysis and Reporting System (CDARS), a territory‐wide database that centralizes patient information from individual local hospitals to establish comprehensive medical data, including clinical characteristics, disease diagnosis, laboratory results, and drug treatment details. The system has been previously used by both our team and other teams in Hong Kong to conduct population‐based cohort studies,^18^, ^19^ including those on diabetes mellitus.^20^, ^21^

Clinical and biochemical data were extracted from CDARS for the present study. Patients' demographics include sex and age of initial drug use (baseline). Prior comorbidities before initial drug use were extracted, including diabetes with chronic complication, diabetes without chronic complication, gout, hypertension, ischaemic heart disease, liver diseases, peripheral vascular disease, renal diseases, stroke/transient ischaemic attack, atrial fibrillation, ventricular tachycardia (VT)/ventricular fibrillation (VF)/aborted sudden cardiac death (SCD), anaemia, overweight, and cancer. Charlson's standard comorbidity index was also calculated. Mortality was recorded using the *International Classification of Diseases Tenth Edition* (ICD‐10) coding, whilst the study outcomes and comorbidities were documented in CDARS under ICD‐9 codes. The ICD codes used to search for diagnoses and outcomes are shown in Supporting Information, *Table S1*.

Non‐SGLT2I/DPP4I medications were also extracted, including metformin, sulphonylurea, insulin, acarbose, thiazolidinedione, glucagon‐like peptide‐1 receptor agonists, and statins and fibrates. A limited number of enrolled patients have been prescribed calcium channel blockers; thus, they were not considered. Baseline laboratory data were extracted. Subclinical biomarkers were calculated accordingly, including neutrophil‐to‐lymphocyte ratio, platelet‐to‐lymphocyte ratio, neutrophil‐to‐high‐density lipoprotein ratio, lymphocyte‐to‐high‐density lipoprotein ratio, lymphocyte‐to‐low‐density lipoprotein ratio, low‐density lipoprotein ratio‐to‐high‐density lipoprotein ratio, total cholesterol‐to‐high‐density lipoprotein ratio, triglyceride‐glucose index, bilirubin‐to‐albumin ratio, protein‐to‐creatinine ratio, and prognostic nutritional index.

Standard deviation (SD) was calculated for glycaemic and lipid profile parameters once there are at least three examinations for each patient since initial drug exposure of SGLT2I or DPP4I. We also calculated more specific variability measures for HbA1c and fasting glucose profiles including SD, SD/initial, coefficient of variation (CV), and variability independent of mean as listed in Supporting Information, *Table* *S2*.

### Outcomes and statistical analysis

The study outcomes are new‐onset HF, and new‐onset MI, cardiovascular mortality, and all‐cause mortality as defined by the first incidence of ICD‐9 codes of these adverse events (Supporting Information, *Table S1*). Mortality data were obtained from the Hong Kong Death Registry, a population‐based official government registry with the registered death records of all Hong Kong citizens linked to CDARS. ICD‐10 codes I00–I09, I11, I13, and I20–I51 were used to identify cardiovascular mortality. Descriptive statistics are used to summarize baseline clinical and biochemical characteristics of patients with SGLT2I and DPP4I use. For baseline clinical characteristics, the continuous variables were presented as median (95% confidence interval [CI]/interquartile range [IQR]) or mean (SD) and the categorical variables were presented as total number (percentage). Continuous variables were compared using the two‐tailed Mann–Whitney *U* test, whilst the two‐tailed *χ* ^2^ test with Yates' correction was used to test 2 × 2 contingency data. Univariable Cox regression was used to identify significant predictors for the primary and secondary outcomes. Propensity score matching was performed to generate control of SGLT2I users to compare against DPP4I users in a 1:1 ratio based on baseline age, sex, prior comorbidities, and non‐SGLT2I/DPP4I medications using nearest neighbour matching strategy.

Multivariable Cox models adjusting for significant risk factors of demographics, past comorbidities, non‐SGLT2I/DPP4I medications, subclinical biomarkers, HbA1c, and fasting glucose to identify the treatment effects of SGLT2I vs. DPP4I on the mentioned adverse outcomes. Cause‐specific and subdistribution hazard models were conducted to consider possible competing risks. Lastly, subgroup analyses were done on age (≤65 and >65 years) and sex on drug exposure effects. A standardized mean difference (SMD) of no <0.2 between the treatment groups post‐weighting was considered negligible. The hazard ratio (HR), 95% CI, and *P*‐value were reported. Statistical significance is defined as *P*‐value < 0.05. The statistical analysis was performed with RStudio software (Version 1.1.456) and Python (Version 3.6).

## Results

### Baseline characteristics

**Figure 1:**
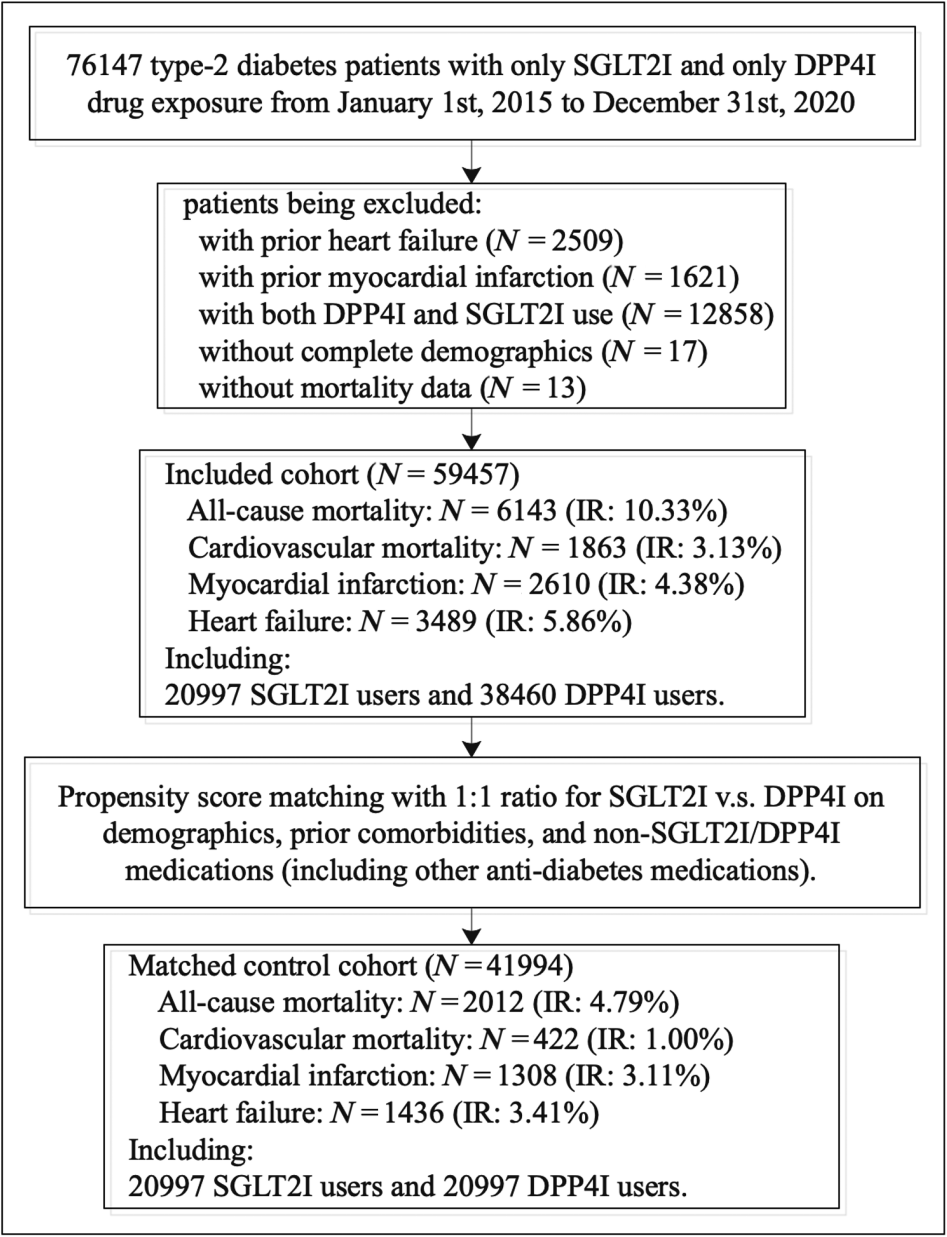
Flowchart of data processing. DPP4I, dipeptidyl peptidase‐4 inhibitors; IR, incidence rate; SGLT2I, sodium‐glucose cotransporter‐2 inhibitors.

In this study, patients with type 2 diabetes mellitus and use of either SGLT2I or DPP4I from 1 January 2015 to 31 December 2020 were included (*Table* *1*). Patients with the use of both classes, or with prior HF diagnoses or admissions due to HF or with anti‐HF drugs (e.g. beta‐blockers for HF and diuretics for HF), were excluded. After exclusion, 41 994 patients (58.89% males, median admission age at 58 years old, IQR: 51.2–65.3) fulfilled the eligibility criteria in the study cohort for subsequent analysis (*Figure* *1*). The study cohort has a median follow‐up duration of 5.6 years (IQR: 5.32–5.82). Propensity score matching (1:1) between SGLT2I and DPP4I users using the nearest neighbour search strategy with a 0.1 caliper was performed (Supporting Information, *Figure S1*). Bootstrapping procedures were performed for propensity matching estimates, and the estimations of bootstrapped standard error (replications = 50) were <0.001. Together, these indicated no significant confounding characteristics remained significant after propensity matching.

**Table 1:** Baseline and clinical characteristics of patients with SGLT2I vs. DPP4I uses before and after propensity score matching (1:1)

Characteristics	Before matching			SMD	After matching			SMD
	All (*N* = 59 457) Mean (SD); *N* or count (%)	SGLT2I users (*N* = 20 997) Mean (SD); *N* or count (%)	DPP4I users (*N* = 38 460) Mean (SD); *N* or count (%)		All (*N* = 41 994) Mean (SD); *N* or count (%)	SGLT2I users (*N* = 20 997) Mean (SD); *N* or count (%)	DPP4I users (*N* = 20 997) Mean (SD); *N* or count (%)	
***Outcomes***								
All‐cause mortality	6143 (10.33%)	527 (2.50%)	5616 (14.60%)	0.44*	2674 (6.36%)	527 (2.50%)	2147 (10.22%)	0.32*
Cardiovascular mortality	1863 (3.13%)	105 (0.50%)	1758 (4.57%)	0.26*	538 (1.28%)	105 (0.50%)	433 (2.06%)	0.14
Myocardial infarction	2610 (4.38%)	631 (3.00%)	1979 (5.14%)	0.11	1649 (3.92%)	631 (3.00%)	1018 (4.84%)	0.1
Heart failure	3489 (5.86%)	638 (3.03%)	2851 (7.41%)	0.2	1809 (4.30%)	638 (3.03%)	1171 (5.57%)	0.13
***Demographics***								
Male gender	32 686 (54.97%)	12 403 (59.07%)	20 283 (52.73%)	0.13	26 527 (63.16%)	12 403 (59.07%)	14 124 (67.26%)	0.17
Baseline age, years	62.9 (12.8); *n* = 59 457	57.5 (11.3); *n* = 20 997	65.8 (12.7); *n* = 38 460	0.69*	61.1 (13.2); *n* = 41 994	57.5 (11.3); *n* = 20 997	64.6 (14.0); *n* = 20 997	0.56*
<50	8572 (14.41%)	4700 (22.38%)	3872 (10.06%)	0.34*	6834 (16.27%)	4700 (22.38%)	2134 (10.16%)	0.34*
[50–60]	16 503 (27.75%)	7545 (35.93%)	8958 (23.29%)	0.28*	14 965 (35.63%)	7545 (35.93%)	7420 (35.33%)	0.01
[60–70]	17 357 (29.19%)	6167 (29.37%)	11 190 (29.09%)	0.01	10 146 (24.16%)	6167 (29.37%)	3979 (18.95%)	0.25*
[70–80]	10 748 (18.07%)	2119 (10.09%)	8629 (22.43%)	0.34*	5394 (12.84%)	2119 (10.09%)	3275 (15.59%)	0.17
>80	6282 (10.56%)	469 (2.23%)	5813 (15.11%)	0.47*	4658 (11.09%)	469 (2.23%)	4189 (19.95%)	0.59*
***Past comorbidities***								
Charlson's standard comorbidity index	2.0 (1.4); *n* = 59 457	1.5 (1.2); *n* = 20 997	2.3 (1.5); *n* = 38 460	0.62*	1.8 (1.4); *n* = 41 994	1.5 (1.2); *n* = 20 997	2.1 (1.5); *n* = 20 997	0.5*
Diabetes with chronic complication	597 (1.00%)	227 (1.08%)	370 (0.96%)	0.01	454 (1.08%)	227 (1.08%)	227 (1.08%)	<0.01
Diabetes without chronic complication	1021 (1.71%)	441 (2.10%)	580 (1.50%)	0.04	872 (2.07%)	441 (2.10%)	431 (2.05%)	<0.01
Gout	1463 (2.46%)	421 (2.00%)	1042 (2.70%)	0.05	838 (1.99%)	421 (2.00%)	417 (1.98%)	<0.01
Hyperlipidaemia	1531 (2.57%)	727 (3.46%)	804 (2.09%)	0.08	1445 (3.44%)	727 (3.46%)	718 (3.41%)	<0.01
Hypertension	13 262 (22.30%)	4684 (22.30%)	8578 (22.30%)	<0.01	9416 (22.42%)	4684 (22.30%)	4732 (22.53%)	0.01
Hypoglycaemia	442 (0.74%)	50 (0.23%)	392 (1.01%)	0.1	100 (0.23%)	50 (0.23%)	50 (0.23%)	<0.01
Ischaemic heart disease	4069 (6.84%)	1962 (9.34%)	2107 (5.47%)	0.15	3837 (9.13%)	1962 (9.34%)	1875 (8.92%)	0.01
Liver diseases	1278 (2.14%)	634 (3.01%)	644 (1.67%)	0.09	1257 (2.99%)	634 (3.01%)	623 (2.96%)	<0.01
Peripheral vascular disease	393 (0.66%)	98 (0.46%)	295 (0.76%)	0.04	196 (0.46%)	98 (0.46%)	98 (0.46%)	<0.01
Renal diseases	972 (1.63%)	105 (0.50%)	867 (2.25%)	0.15	210 (0.50%)	105 (0.50%)	105 (0.50%)	<0.01
Stroke/transient ischaemic attack	1842 (3.09%)	509 (2.42%)	1333 (3.46%)	0.06	1015 (2.41%)	509 (2.42%)	506 (2.40%)	<0.01
Atrial fibrillation	1017 (1.71%)	325 (1.54%)	692 (1.79%)	0.02	649 (1.54%)	325 (1.54%)	324 (1.54%)	<0.01
VT/VF/aborted SCD	64 (0.10%)	29 (0.13%)	35 (0.09%)	0.01	58 (0.13%)	29 (0.13%)	29 (0.13%)	<0.01
Anaemia	2229 (3.74%)	456 (2.17%)	1773 (4.60%)	0.14	910 (2.16%)	456 (2.17%)	454 (2.16%)	<0.01
Overweight	395 (0.66%)	324 (1.54%)	71 (0.18%)	0.15	644 (1.53%)	324 (1.54%)	320 (1.52%)	<0.01
Cancer	1611 (2.70%)	427 (2.03%)	1184 (3.07%)	0.07	851 (2.02%)	427 (2.03%)	424 (2.01%)	<0.01
***Medications***								
SGLT2I vs. DPP4I	20 997 (35.31%)	20 997 (100.00%)	0 (0.00%)	inf*	20 997 (50.00%)	20 997 (100.00%)	0 (0.00%)	inf*
SGLT2I frequency	7.2 (9.7); *n* = 20 997	7.2 (9.7); *n* = 20 997	‐	‐	7.2 (9.7); *n* = 20 997	7.2 (9.7); *n* = 20 997	‐	‐
DPP4I frequency	5.3 (7.4); *n* = 38 460	‐	5.3 (7.4); *n* = 38 460	‐	6.8 (7.0); *n* = 20 997	‐	6.8 (7.0); *n* = 20 997	‐
SGLT2I duration, days	527.9 (670.1); *n* = 20 997	527.9 (670.1); *n* = 20 997	‐	‐	527.9 (670.1); *n* = 20 997	527.9 (670.1); *n* = 20 997	‐	‐
DPP4I duration, days	490.7 (416.9); *n* = 38 460	‐	490.7 (416.9); *n* = 38 460	‐	449.9 (347.5); *n* = 20 997	‐	449.9 (347.5); *n* = 20 997	‐
Metformin	53 053 (89.22%)	19 492 (92.83%)	33 561 (87.26%)	0.19	38 984 (92.83%)	19 492 (92.83%)	19 492 (92.83%)	<0.01
Sulphonylurea	45 591 (76.67%)	14 675 (69.89%)	30 916 (80.38%)	0.24*	29 798 (70.95%)	14 675 (69.89%)	15 123 (72.02%)	0.05
Insulin	29 710 (49.96%)	10 746 (51.17%)	18 964 (49.30%)	0.04	21 592 (51.41%)	10 746 (51.17%)	10 846 (51.65%)	0.01
Acarbose	1505 (2.53%)	844 (4.01%)	661 (1.71%)	0.14	1666 (3.96%)	844 (4.01%)	822 (3.91%)	0.01
Thiazolidinediones	11 448 (19.25%)	5960 (28.38%)	5488 (14.26%)	0.35*	11 590 (27.59%)	5960 (28.38%)	5630 (26.81%)	0.04
Glucagon‐like peptide‐1 receptor agonists	1693 (2.84%)	1521 (7.24%)	172 (0.44%)	0.36*	2823 (6.72%)	1521 (7.24%)	1302 (6.20%)	0.04
Statins and fibrates	28 231 (47.48%)	15 189 (72.33%)	13 042 (33.91%)	0.83*	29 651 (70.60%)	15 189 (72.33%)	14 462 (68.87%)	0.08
***Complete blood counts***								
Haemoglobin, g/dL	13.1 (1.8); *n* = 30 341	13.7 (1.5); *n* = 12 023	12.7 (1.9); *n* = 18 318	0.57*	12.9 (1.9); *n* = 23 614	13.7 (1.5); *n* = 12 023	12.1 (1.9); *n* = 11 591	0.94*
Mean corpuscular volume, fL	87.2 (7.6); *n* = 29 824	86.7 (7.1); *n* = 11 897	87.6 (7.8); *n* = 17 927	0.12	86.0 (7.7); *n* = 23 482	86.7 (7.1); *n* = 11 897	85.4 (8.2); *n* = 11 585	0.17
Eosinophil, ×10^9^/L	0.2 (0.3); *n* = 23 887	0.21 (0.19); *n* = 9293	0.22 (0.28); *n* = 14 594	0.02	0.3 (0.2); *n* = 18 510	0.2 (0.2); *n* = 9293	0.3 (0.2); *n* = 9217	0.34*
Lymphocyte, ×10^9^/L	2.0 (0.9); *n* = 23 910	2.2 (0.9); *n* = 9298	1.9 (0.9); *n* = 14 612	0.29*	2.0 (0.8); *n* = 18 515	2.2 (0.9); *n* = 9298	1.8 (0.6); *n* = 9217	0.46*
Neutrophil, ×10^9^/L	5.3 (2.8); *n* = 23 910	5.1 (2.4); *n* = 9298	5.5 (3.1); *n* = 14 612	0.13	4.9 (2.2); *n* = 18 515	5.1 (2.4); *n* = 9298	4.6 (1.9); *n* = 9217	0.23*
White cell count, ×10^9^/L	8.0 (3.0); *n* = 29 836	7.96 (2.62); *n* = 11 905	8.03 (3.24); *n* = 17 931	0.03	7.7 (2.4); *n* = 23 490	8.0 (2.6); *n* = 11 905	7.4 (2.1); *n* = 11 585	0.23*
Mean cell haemoglobin, pg	29.4 (3.0); *n* = 29 824	29.2 (2.9); *n* = 11 897	29.6 (3.1); *n* = 17 927	0.14	28.9 (3.0); *n* = 23 482	29.2 (2.9); *n* = 11 897	28.7 (3.2); *n* = 11 585	0.17
Platelet, ×10^9^/L	241.3 (72.4); *n* = 29 834	246.2 (68.1); *n* = 11 903	238.1 (74.9); *n* = 17 931	0.11	249.5 (65.3); *n* = 23 488	246.2 (68.1); *n* = 11 903	252.8 (62.3); *n* = 11 585	0.1
Red cell count, ×10^12^/L	4.5 (0.7); *n* = 29 824	4.7 (0.6); *n* = 11 897	4.4 (0.7); *n* = 17 927	0.57*	4.5 (0.7); *n* = 23 482	4.7 (0.6); *n* = 11 897	4.3 (0.7); *n* = 11 585	0.72*
***Liver and renal function tests***								
Potassium, mmol/L	4.4 (0.5); *n* = 49 049	4.3 (0.4); *n* = 17 701	4.4 (0.5); *n* = 31 348	0.14	4.3 (0.5); *n* = 35 665	4.31 (0.43); *n* = 17 701	4.3 (0.57); *n* = 17 964	0.02
Albumin, g/L	41.7 (4.0); *n* = 37 392	42.5 (3.3); *n* = 15 014	41.1 (4.3); *n* = 22 378	0.38*	41.4 (3.8); *n* = 27 777	42.5 (3.3); *n* = 15 014	40.1 (4.0); *n* = 12 763	0.66*
Sodium, mmol/L	139.3 (3.0); *n* = 49 074	139.2 (2.7); *n* = 17 704	139.3 (3.1); *n* = 31 370	0.05	138.7 (2.8); *n* = 35 664	139.2 (2.7); *n* = 17 704	138.2 (2.9); *n* = 17 960	0.34*
Urea, mmol/L	6.6 (3.5); *n* = 49 058	5.7 (2.0); *n* = 17 696	7.1 (4.1); *n* = 31 362	0.43*	6.4 (2.9); *n* = 35 645	5.7 (2.0); *n* = 17 696	7.1 (3.5); *n* = 17 949	0.5*
Protein, g/L	73.9 (5.5); *n* = 35 190	74.4 (4.9); *n* = 14 192	73.5 (5.9); *n* = 20 998	0.17	73.1 (5.3); *n* = 26 754	74.4 (4.9); *n* = 14 192	71.5 (5.3); *n* = 12 562	0.56*
Creatinine, μmol/L	94.8 (76.8); *n* = 49 203	78.1 (28.5); *n* = 17 733	104.2 (92.4); *n* = 31 470	0.38*	87.3 (45.8); *n* = 35 695	78.1 (28.5); *n* = 17 733	96.4 (56.7); *n* = 17 962	0.41*
Alkaline phosphatase, U/L	77.0 (32.7); *n* = 37 508	73.7 (25.8); *n* = 15 019	79.2 (36.5); *n* = 22 489	0.17	72.1 (24.8); *n* = 27 808	73.7 (25.8); *n* = 15 019	70.2 (23.4); *n* = 12 789	0.14
Aspartate transaminase, U/L	28.0 (48.4); *n* = 14 801	28.5 (27.9); *n* = 6004	27.7 (58.4); *n* = 8797	0.02	27.4 (29.2); *n* = 9431	28.5 (27.9); *n* = 6004	25.6 (31.1); *n* = 3427	0.1
Alanine transaminase, U/L	29.1 (33.8); *n* = 31 957	32.6 (29.7); *n* = 12 805	26.8 (36.2); *n* = 19 152	0.18	27.2 (25.4); *n* = 26 576	32.6 (29.7); *n* = 12 805	22.1 (19.4); *n* = 13 771	0.42*
Bilirubin, μmol/L	11.2 (7.1); *n* = 37 319	11.4 (6.2); *n* = 14 986	11.0 (7.6); *n* = 22 333	0.05	11.1 (6.2); *n* = 27 757	11.4 (6.2); *n* = 14 986	10.7 (6.2); *n* = 12 771	0.11
***Lipid and glucose profiles***								
Triglyceride, mmol/L	1.7 (1.5); *n* = 46 180	1.8 (1.7); *n* = 16 999	1.7 (1.3); *n* = 29 181	0.1	1.7 (1.5); *n* = 31 403	1.8 (1.7); *n* = 16 999	1.6 (1.1); *n* = 14 404	0.15
Low‐density lipoprotein, mmol/L	2.4 (0.8); *n* = 45 409	2.4 (0.81); *n* = 16 707	2.39 (0.8); *n* = 28 702	0.01	2.3 (0.7); *n* = 30 955	2.4 (0.8); *n* = 16 707	2.3 (0.6); *n* = 14 248	0.17
High‐density lipoprotein, mmol/L	1.2 (0.3); *n* = 46 115	1.17 (0.31); *n* = 16 971	1.22 (0.34); *n* = 29 144	0.15	1.2 (0.3); *n* = 31 373	1.17 (0.31); *n* = 16 971	1.16 (0.36); *n* = 14 402	0.03
Total cholesterol, mmol/L	4.3 (1.0); *n* = 46 221	4.4 (1.0); *n* = 17 016	4.3 (1.0); *n* = 29 205	0.01	4.3 (0.9); *n* = 31 423	4.4 (1.0); *n* = 17 016	4.1 (0.8); *n* = 14 407	0.23*
Glucose, mmol/L	8.9 (3.9); *n* = 43 596	9.2 (3.6); *n* = 16 077	8.7 (4.0); *n* = 27 519	0.12	9.0 (4.8); *n* = 30 395	9.2 (3.6); *n* = 16 077	8.8 (5.8); *n* = 14 318	0.09

DPP4I, dipeptidyl peptidase‐4 inhibitor; SCD, sudden cardiac death; SD, standard deviation; SGLT2I, sodium‐glucose cotransporter‐2 inhibitor; SMD, standardized mean difference; VF, ventricular fibrillation; VT, ventricular tachycardia.

* SMD ≥ 0.2.

### Significant predictors of the study outcomes

**Figure 2:**
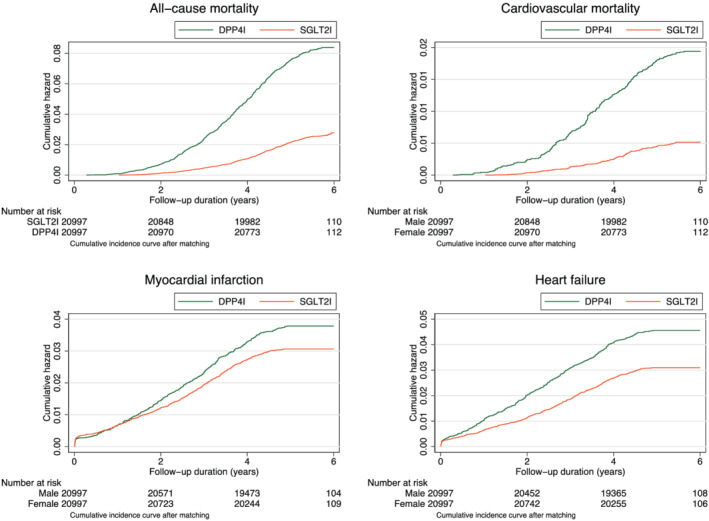
Cumulative incidence curves for heart failure, myocardial infarction, cardiovascular mortality, and all‐cause mortality stratified by SGLT2I or DPP4I use in the matched cohort. DPP4I, dipeptidyl peptidase‐4 inhibitors; SGLT2I, sodium‐glucose cotransporter‐2 inhibitors.

The cumulative incidence curves for new‐onset HF, MI, cardiovascular mortality, and all‐cause mortality stratified by SGLT2I or DPP4I use for the matched cohort are shown in *Figure* *2*. Lower incidences of all of these outcomes were observed for SGLT2I users compared with DPP4I users. Univariable Cox regression was applied to identify significant predictors of the study outcomes (Supporting Information, *Tables* *S2* and *S3*). In the matched cohort, SGLT2I use was associated with significantly lower risks of new‐onset HF (HR: 0.52, 95% CI: [0.48, 0.58], *P* < 0.0001), new‐onset MI (HR: 0.60, 95% CI: [0.54, 0.66], *P* < 0.0001), cardiovascular mortality (HR: 0.23, 95% CI: [0.18, 0.28], *P* < 0.0001), and all‐cause mortality (HR: 0.23, 95% CI: [0.21, 0.26], *P* < 0.0001). Multivariable Cox models were developed adjusting for significant demographics, past comorbidities, and medications (*Table* *2*). SGLT2I use remained a significant predictor of all four study outcomes (HR < 1, *P* < 0.001).

**Table 2:** Multivariable Cox regression for heart failure, myocardial infarction, cardiovascular mortality, and all‐cause mortality in the matched cohort

Adverse outcomes	Model 1 HR [95% CI]; *P*‐value	Model 2 HR [95% CI]; *P*‐value	Model 3 HR [95% CI]; *P*‐value	
All‐cause mortality	0.30 [0.28–0.34]; <0.0001***	0.30 [0.27–0.33]; <0.0001***	0.26 [0.24–0.29]; <0.0001***	
Cardiovascular mortality	0.65 [0.35–0.86]; 0.0057**	0.75 [0.59–0.94]; 0.0128*	0.67 [0.53–0.84]; 0.0005***	
Myocardial infarction	0.81 [0.73–0.90]; 0.0001***	0.81 [0.73–0.91]; 0.0002***	0.81 [0.73–0.90]; 0.0001***	
Heart failure	0.79 [0.71–0.87]; <0.0001***	0.77 [0.69–0.85]; <0.0001***	0.73 [0.66–0.81]; <0.0001***	

CI, confidence interval; HR, hazard ratio.

Model 1 adjusted for significant demographics. Model 2 adjusted for significant demographics and past comorbidities. Model 3 adjusted for significant demographics, past comorbidities, and non‐sodium‐glucose cotransporter‐2 inhibitor/dipeptidyl peptidase‐4 inhibitor medications.

* *P* ≤ 0.05.

** *P* ≤ 0.01.

*** *P* ≤ 0.001.

To evaluate the predictiveness of the models, different sensitivity analyses were performed. Firstly, a 1 year lag time between treatment initiation and study outcomes was applied (Supporting Information, *Table* *S4*). Secondly, competing risk analyses using cause‐specific hazard models and subdistribution hazard models were applied (Supporting Information, *Table* *S5*). Thirdly, different propensity score approaches were used to evaluate the effects of the matching approach on the analysis, including propensity score stratification, inverse probability of treatment weighting (IPTW), and stable inverse probability of treatment weighting (SIPTW) (Supporting Information, *Table* *S6*). All of these analyses demonstrated that SGLT2I use was associated with lower risks of new‐onset HF, MI, cardiovascular mortality, and all‐cause mortality.

## Discussion

The main finding of the present study is that using DPP4I as a reference, SGLT2I use was associated with a lower risk of new‐onset HF and MI, cardiovascular mortality, and all‐cause mortality.

Our findings are largely consistent with existing studies. A network meta‐analysis of 236 trials has reported the superior cardiovascular protective effects of SGLT2I against DPP4I when users of either medication are compared against the control group. However, the control groups were not matched and no direct comparison was made.^14^ A recent study evaluating the cardiovascular effects of SGLT2I and DPP4I amongst cardiorenal disease‐free diabetic patients shows that SGLTI users have a lower risk of HF.^17^ However, this study found the effect of SGLT2I on the prevention of acute MI to be neutral, which may be explained by the inherent difference between patients with renal failure and the general population. With a structured follow‐up and close monitoring, patients with renal failure would have their cardiovascular risk factors optimized as a part of their disease management. Moreover, recent meta‐analyses have reported the benefits of SGLT2I in preventing cardiac remodelling in HF patients regardless of glycaemic status^22^ and reducing major clinical events in patients with established HF,^23^ with a neutral effect on arrhythmic outcomes.^24^ Furthermore, a meta‐analysis including more than 34 000 patients found that the protective effect of SGLT2I on major cardiovascular adverse events of atherosclerotic origin is limited to patients with established atherosclerotic disease.^25^ The difference in the proportion of patients with established atherosclerosis may explain the different effects of SGLT2I on MI observed. The present study demonstrates that the cardiovascular beneficent effects of SGLT2I persist in diabetic patients with pre‐existing cardiovascular impairment.

There are several hypotheses for the mechanisms underlying the cardiovascular‐protective effects of SGLT2I. First of all, the modulatory effect of SGLT2I on the proximal tubules results in glucosuria and natriuresis, thus lowering the preload and the resulting stress on the ventricles.^26^ It is speculated that SGLT2I has a unique effect of selectively contracting interstitial fluid specifically, without affecting the intravascular volume, thus particularly useful in the prevention of HF.^27^ The hypothesis is supported by studies comparing the vascular effects of dapagliflozin and bumetanide, where dapagliflozin has been shown to have little effect on the intravascular volume.^28^, ^29^

Moreover, inhibition of the sodium‐hydrogen ion exchanger in the myocardium, which is activated under HF to increase intracytoplasmic sodium and calcium level, was also hypothesized to be a part of the underlying mechanism.^30^, ^31^ However, because SGLT2 receptors are absent in the heart, the exact inhibitory mechanism remains unclear. Other hypotheses on the anti‐fibrosis and adipokine‐reducing effects, which are effective against both HF and MI, suggest that the cardiovascular‐protective effects of SGLT2I may involve multiple biochemical pathways and thus protect against different cardiovascular diseases.^27^, ^32^

The multiple processes involved in the cardiovascular‐protective effect of SGLTI may also explain its superior outcome against DPP4I. Whilst previous studies reported the benefits of SGLT2I on cardiovascular health are mainly attributed to its protection against HF, a recent territory‐wide study has shown that SGLT2I users have a lower incidence of new‐onset atrial fibrillation than DPP4I users, which supports the lower cardiovascular and all‐cause mortality reported in the present study.^16^ This may be attributed to the anti‐fibrotic effects of SGLT2I, because atrial remodelling and fibrosis are common pathogenic pathways of atrial fibrillation.^33^ The favourable pleiotropic effects of SGLT2I may also improve the patients' cardiometabolic risk, thus further lowering their MI and cardiovascular mortality risk.^15^ It should be noted that randomized controlled trials have reported that saxagliptin increases the hospitalization rate for HF, despite having a neutral effect on the occurrence of major cardiovascular adverse effects.^34^, ^35^ Because the present study focuses on the incident occurrence of HF and MI, patients on saxagliptin were kept in the study. Amongst the 69 521 patients with type 2 diabetes mellitus, there were in total 353 patients who used saxagliptin use with a low incidence rate of 0.51%.

### Limitations

There are several limitations to the present study. Firstly, inherent information bias with a risk of under‐coding and coding errors should be noted, given its observational and retrospective nature. However, the difference in patient characteristics, past comorbidities, and other medication usages between SGLT4I/DPP4I users and controls was addressed through matching using propensity scores, although residual bias may remain. There are also patients with missing data for the laboratory parameters because not all blood tests were routinely performed for all. Moreover, we were unable to access important lifestyle predictors for cardiovascular adverse events, such as body mass index, smoking, and alcoholism. Thirdly, coding for clinical diagnoses of HF was used but echocardiographic data are not coded in the administrative database, and therefore, different types of HF based on ejection fraction could not be examined. Finally, DPP4I use is associated with an increased risk of HF compared with placebo, and therefore, this study could not distinguish between whether gliptins cause HF and whether SGLT2I reduce HF.

## Conclusions

SGLT2 inhibitors are protective against adverse cardiovascular events including new‐onset HF, MI, cardiovascular mortality, and all‐cause mortality. The prescription of SGLT2I is preferred when taken into consideration individual cardiovascular and metabolic risk profiles in addition to drug–drug interactions.

## Conflict of interest

None declared.

## Funding

None.

## Author contributions

Jiandong Zhou and Sharen Lee: conception of study and literature search, preparation of figures, study design, data collection, data contribution, statistical analysis, data interpretation, manuscript drafting, and critical revision of the manuscript.

Keith Sai Kit Leung, Abraham Ka Chung Wai, Tong Liu, Ying Liu, Dong Chang, Wing Tak Wong, Ian Chi Kei Wong, and Bernard Man Yung Cheung: conception of study and literature search, data collection, data contribution, critical revision of the manuscript, and study supervision.

Qingpeng Zhang and Gary Tse: conception of study and literature search, study design, data collection, data analysis, data contribution, manuscript drafting, critical revision of manuscript, and study supervision.

## Supporting information


**Figure S1.** Propensity score matching comparisons for SGLT2I v.s. DPP4I before and after 1:1 matching with nearest neighbor search strategy using a caliper of 0.1.
**Table S1.** ICD‐9 codes for diagnoses and ICD‐10 codes for outcomes.
**Table S2.** Univariable Cox regression to identify significant predictors of heart failure and myocardial infarction before and after 1:1 matching.
* for *P* ≤ 0.05, ** for *P* ≤ 0.01, *** for *P* ≤ 0.001; HR: hazard ratio; CI: confidence interval; SD: standard deviation; SCD: sudden cardiac death; VF: ventricular fibrillation; VT: ventricular tachycardia; SGLT2I: sodium glucose cotransporter‐2 inhibitor; DPP4I: dipeptidyl peptidase‐4 inhibitor; CV: coefficient of variation.
**Table S3.** Univariable Cox regression to identify significant predictors of cardiovascular mortality and all‐cause mortality before and after 1:1 matching.
* for *P* ≤ 0.05, ** for *P* ≤ 0.01, *** for *P* ≤ 0.001; HR: hazard ratio; CI: confidence interval; SD: standard deviation; SCD: sudden cardiac death; VF: ventricular fibrillation; VT: ventricular tachycardia; SGLT2I: sodium glucose cotransporter‐2 inhibitor; DPP4I: dipeptidyl peptidase‐4 inhibitor; CV: coefficient of variation.
**Table S4.** Sensitivity analysis 1: Multivariable Cox models with a one‐year lag time.
**Table S5.** Sensitivity analysis 2: Competing risk analyses.
**Table S6.** Sensitivity analysis 3: Different propensity score approaches.
