# Supplementary material for: Incident heart failure and myocardial infarction in sodium‐glucose cotransporter‐2 vs. dipeptidyl peptidase‐4 inhibitor users
Source: ESC Heart Fail. 2022 Feb 7;9(2):1388–99. doi: 10.1002/ehf2.13830 (PMC8934922; doi:10.1002/ehf2.13830)
Supplement: Supplementary file 1 — Figure S1. Propensity score matching comparisons for SGLT2I v.s. DPP4I before and after 1:1 matching with nearest neighbor search strategy using a caliper of 0.1. Table S1. ICD‐9 codes for diagnoses and ICD‐10 codes for outcomes. Table S2. Univariable Cox regression to identify significant predictors of heart failure and myocardial infarction before and after 1:1 matching. * for P ≤ 0.05, ** for P ≤ 0.01, *** for P ≤ 0.001; HR: hazard ratio; CI: confidence interval; SD: standard deviation; SCD: sudden cardiac death; VF: ventricular fibrillation; VT: ventricular tachycardia; SGLT2I: sodium glucose cotransporter‐2 inhibitor; DPP4I: dipeptidyl peptidase‐4 inhibitor; CV: coefficient of variation. Table S3. Univariable Cox regression to identify significant predictors of cardiovascular mortality and all‐cause mortality before and after 1:1 matching. * for P ≤ 0.05, ** for P ≤ 0.01, *** for P ≤ 0.001; HR: hazard ratio; CI: confidence interval; SD: standard deviation; SCD: sudden cardiac death; VF: ventricular fibrillation; VT: ventricular tachycardia; SGLT2I: sodium glucose cotransporter‐2 inhibitor; DPP4I: dipeptidyl peptidase‐4 inhibitor; CV: coefficient of variation. Table S4. Sensitivity analysis 1: Multivariable Cox models with a one‐year lag time. Table S5. Sensitivity analysis 2: Competing risk analyses. Table S6. Sensitivity analysis 3: Different propensity score approaches. [file EHF2-9-1388-s001.docx]

**Supplementary Appendix**

**Supplementary Figure 1. Propensity score matching comparisons for SGLT2I v.s. DPP4I before and after 1:1 matching with nearest neighbor search strategy using a caliper of 0.1**

**Supplementary Table 1. ICD-9 codes for diagnoses and ICD-10 codes for outcomes.**

| Diabetes mellitus 250 250.01 250.02 250.03 250.1 250.11 250.12 250.13 250.2 250.21 250.22 250.23 250.3 250.31 250.32 250.33 250.4 250.41 250.42 250.43 250.5 250.51 250.52 250.53 250.6 250.61 250.62 250.63 250.7 250.71 250.72 250.73 250.8 250.81 250.82 250.83 250.9 250.91 250.92 250.93 |
| --- |
| Renal diseases 582 582 582.1 582.2 582.4 582.8 582.81 582.89 582.9 583 583 583.1 583.2 583.4 583.6 583.7 585 585.1 585.2 585.3 585.4 585.5 585.6 585.9 586 588 588 588.1 588.8 588.81 588.89 588.9 |
| Acute myocardial infarction 410 410.01 410.02 410.1 410.11 410.12 410.2 410.21 410.22 410.3 410.31 410.32 410.4 410.41 410.42 410.5 410.51 410.52 410.6 410.61 410.62 410.7 410.71 410.72 410.8 410.81 410.82 410.9 410.91 410.92 |
| Hypertension 401 401.1 401.9 402 402.01 402.1 402.11 402.9 402.91 403 403.01 403.1 403.11 403.9 403.91 404 404.01 404.02 404.03 404.1 404.11 404.12 404.13 404.9 404.91 404.92 404.93 405 405.01 405.09 405.1 405.11 405.19 405.9 405.91 405.99 437.2 |
| Heart failure 428 428 428.1 428.2 428.2 428.21 428.22 428.23 428.3 428.3 428.31 428.32 428.33 428.4 428.4 428.41 428.42 428.43 428.9 398.91 402.01 402.11 402.91 404.01 404.03 404.11 404.13 404.91 404.93 |
| Atrial fibrillation 427.31 429.4 |
| Liver diseases 456 456.1 456.2 572.2 572.3 572.4 572.8 571.4 571.5 571.6 |
| Chronic obstructive pulmonary disease 490 491 492 493 494 495 496 491.1 491.2 491.21 491.22 491.8 491.9 492.8 493.01 493.02 493.1 493.11 493.12 493.2 493.21 493.22 493.8 493.81 493.82 493.9 493.91 493.92 494.1 495.1 495.2 495.3 495.4 495.5 495.6 495.7 495.8 495.9 |
| PVD 250.7 443.9 443 443.1 443.2 443.21 443.22 443.23 443.24 443.29 443.8 443.81 443.82 443.89 441 443.9 785.4 V43.4 |
| Stroke/transient ischemic attack 435 435.1 435.2 435.3 435.8 435.9 433.81 433.91 434 436 437 437.1 433.31 433.01 434.01 434.1 434.11 434.9 434.91 437.2 437.3 437.4 437.5 437.6 437.7 437.8 437.9 430 431 432 432.1 432.9 |
| Ischemic heart disease 410.01 410.02 410.1 410.11 410.12 410.2 410.21 410.22 410.3 410.31 410.32 410.4 410.41 410.42 410.5 410.51 410.52 410.6 410.61 410.62 410.7 410.71 410.72 410.8 410.81 410.82 410.9 410.91 410.92 411 411.1 411.8 411.81 411.89 413 413.1 413.9 414 414.01 414.02 414.03 414.04 414.05 414.06 414.07 414.1 414.11 414.12 414.19 414.2 414.3 414.4 414.8 414.9 410 412 |
| Cancer 140 140.1 140.3 140.4 140.5 140.6 140.8 140.9 141 141.1 141.2 141.3 141.4 141.5 141.6 141.8 141.9 142 142.1 142.2 142.8 142.9 143 143.1 143.8 143.9 144 144.1 144.8 144.9 145 145.1 145.2 145.3 145.4 145.5 145.6 145.8 145.9 146 146.1 146.2 146.3 146.4 146.5 146.6 146.7 146.8 146.9 147 147.1 147.2 147.3 147.8 147.9 148 148.1 148.2 148.3 148.8 148.9 149 149.1 149.8 149.9 150 150.1 150.2 150.3 150.4 150.5 150.8 150.9 151 151.1 151.2 151.3 151.4 151.5 151.6 151.8 151.9 152 152.1 152.2 152.3 152.8 152.9 153 153.1 153.2 153.3 153.4 153.5 153.6 153.7 153.8 153.9 154 154.1 154.2 154.3 154.8 155 155.1 155.2 156 156.1 156.2 156.8 156.9 157 157.1 157.2 157.3 157.4 157.8 157.9 158 158.8 158.9 159 159.1 159.8 159.9 160 160.1 160.2 160.3 160.4 160.5 160.8 160.9 161 161.1 161.2 161.3 161.8 161.9 162 162.2 162.3 162.4 162.5 162.8 162.9 163 163.1 163.8 163.9 164 164.1 164.2 164.3 164.8 164.9 165 165.8 165.9 170 170.1 170.2 170.3 170.4 170.5 170.6 170.7 170.8 170.9 171 171.2 171.3 171.4 171.5 171.6 171.7 171.8 171.9 172 172.1 172.2 172.3 172.4 172.5 172.6 172.7 172.8 172.9 173 173.01 173.02 173.09 173.1 173.11 173.12 173.19 173.2 173.21 173.22 173.29 173.3 173.31 173.32 173.39 173.4 173.41 173.42 173.49 173.5 173.51 173.52 173.59 173.6 173.61 173.62 173.69 173.7 173.71 173.72 173.79 173.8 173.81 173.82 173.89 173.9 173.91 173.92 173.99 174 174.1 174.2 174.3 174.4 174.5 174.6 174.8 174.9 175 175.9 176 176.1 176.2 176.3 176.4 176.5 176.8 176.9 179 180 180.1 180.8 180.9 181 182 182.1 182.8 183 183.2 183.3 183.4 183.5 183.8 183.9 184 184.1 184.2 184.3 184.4 184.8 184.9 185 186 186.9 187 187.1 187.2 187.3 187.4 187.5 187.6 187.7 187.8 187.9 188 188.1 188.2 188.3 188.4 188.5 188.6 188.7 188.8 188.9 189 189.1 189.2 189.3 189.4 189.8 189.9 190 190.1 190.2 190.3 190.4 190.5 190.6 190.7 190.8 190.9 191 191.1 191.2 191.3 191.4 191.5 191.6 191.7 191.8 191.9 192 192.1 192.2 192.3 192.8 192.9 193 194 194.1 194.3 194.4 194.5 194.6 194.8 194.9 195 195.1 195.2 195.3 195.4 195.5 195.8 200 200.01 200.02 200.03 200.04 200.05 200.06 200.07 200.08 200.1 200.11 200.12 200.13 200.14 200.15 200.16 200.17 200.18 200.2 200.21 200.22 200.23 200.24 200.25 200.26 200.27 200.28 200.3 200.31 200.32 200.33 200.34 200.35 200.36 200.37 200.38 200.4 200.41 200.42 200.43 200.44 200.45 200.46 200.47 200.48 200.5 200.51 200.52 200.53 200.54 200.55 200.56 200.57 200.58 200.6 200.61 200.62 200.63 200.64 200.65 200.66 200.67 200.68 200.7 200.71 200.72 200.73 200.74 200.75 200.76 200.77 200.78 200.8 200.81 200.82 200.83 200.84 200.85 200.86 200.87 200.88 201 201.01 201.02 201.03 201.04 201.05 201.06 201.07 201.08 201.1 201.11 201.12 201.13 201.14 201.15 201.16 201.17 201.18 201.2 201.21 201.22 201.23 201.24 201.25 201.26 201.27 201.28 201.4 201.41 201.42 201.43 201.44 201.45 201.46 201.47 201.48 201.5 201.51 201.52 201.53 201.54 201.55 201.56 201.57 201.58 201.6 201.61 201.62 201.63 201.64 201.65 201.66 201.67 201.68 201.7 201.71 201.72 201.73 201.74 201.75 201.76 201.77 201.78 201.9 201.91 201.92 201.93 201.94 201.95 201.96 201.97 201.98 202 202.01 202.02 202.03 202.04 202.05 202.06 202.07 202.08 202.1 202.11 202.12 202.13 202.14 202.15 202.16 202.17 202.18 202.2 202.21 202.22 202.23 202.24 202.25 202.26 202.27 202.28 202.3 202.31 202.32 202.33 202.34 202.35 202.36 202.37 202.38 202.4 202.41 202.42 202.43 202.44 202.45 202.46 202.47 202.48 202.5 202.51 202.52 202.53 202.54 202.55 202.56 202.57 202.58 202.6 202.61 202.62 202.63 202.64 202.65 202.66 202.67 202.68 202.7 202.71 202.72 202.73 202.74 202.75 202.76 202.77 202.78 202.8 202.81 202.82 202.83 202.84 202.85 202.86 202.87 202.88 202.9 202.91 202.92 202.93 202.94 202.95 202.96 202.97 202.98 203 203.01 203.02 203.1 203.11 203.12 203.8 203.81 203.82 204 204.01 204.02 204.1 204.11 204.12 204.2 204.21 204.22 204.8 204.81 204.82 204.9 204.91 204.92 205 205.01 205.02 205.1 205.11 205.12 205.2 205.21 205.22 205.3 205.31 205.32 205.8 205.81 205.82 205.9 205.91 205.92 206 206.01 206.02 206.1 206.11 206.12 206.2 206.21 206.22 206.8 206.81 206.82 206.9 206.91 206.92 207 207.01 207.02 207.1 207.11 207.12 207.2 207.21 207.22 207.8 207.81 207.82 208 208.01 208.02 208.1 208.11 208.12 208.2 208.21 208.22 208.8 208.81 208.82 208.9 208.91 208.92 196 196.1 196.2 196.3 196.5 196.6 196.8 196.9 197 197.1 197.2 197.3 197.4 197.5 197.6 197.7 197.8 198 198.1 198.2 198.3 198.4 198.5 198.6 198.7 198.8 198.81 198.82 198.89 199 199.1 |
| Obesity 278.01 278 278 |
| Hypertension 401 401.1 401.9 402 402.01 402.1 402.11 402.9 402.91 403 403.01 403.1 403.11 403.9 403.91 404 404.01 404.02 404.03 404.1 404.11 404.12 404.13 404.9 404.91 404.92 404.93 405 405.01 405.09 405.1 405.11 405.19 405.9 405.91 405.99 437.2 |
| Immune mediated enterocolitis 558 558.1 558.2 558.3 558.4 558.41 558.42 558.9 |
| Anemia 280 280.1 280.8 280.9 281 281.1 281.2 281.3 281.4 281.8 281.9 282.2 282.3 282.8 282.9 283 283.1 283.11 283.19 283.2 283.9 284 284.01 284.09 284.1 284.11 284.12 284.19 284.81 284.9 285 285.1 285.2 285.21 285.22 285.29 285.3 285.8 285.9 |
| Overweight 278 278 278 278.01 278.02 278.03 278.1 278.2 278.3 278.4 278.8 |
| Gout 274 274.01 274.02 274.03 274.1 274.11 274.19 274.8 274.81 274.82 274.89 274.9 |
| Cardiovascular mortality I00-I09, I11, I13, I20-I51 |

**Supplementary Table 2. Univariable Cox regression to identify significant predictors of heart failure and myocardial infarction before and after 1:1 matching.**

* for p≤ 0.05, ** for p ≤ 0.01, *** for p ≤ 0.001; HR: hazard ratio; CI: confidence interval; SD: standard deviation; SCD: sudden cardiac death; VF: ventricular fibrillation; VT: ventricular tachycardia; SGLT2I: sodium glucose cotransporter-2 inhibitor; DPP4I: dipeptidyl peptidase-4 inhibitor; CV: coefficient of variation.

| **Characteristics** | **Before matching** |  | **After matching** |  |
| --- | --- | --- | --- | --- |
|  | **Myocardial infarction**  **HR [95% CI];P value** | **Heart failure**  **HR [95% CI];P value** | **Myocardial infarction**  **HR [95% CI];P value** | **Heart failure**  **HR [95% CI];P value** |
| ***Demographics*** |  |  |  |  |
| Male gender | 1.18[1.09-1.27];<0.0001*** | 0.85[0.80-0.91];<0.0001*** | 1.96[1.75-2.20];<0.0001*** | 1.07[0.97-1.17];0.1996 |
| Female gender | 1.0[Reference] | 1.0[Reference] | 1.0[Reference] | 1.0[Reference] |
| Baseline age, years | 1.05[1.05-1.06];<0.0001*** | 1.08[1.07-1.08];<0.0001*** | 1.03[1.03-1.04];<0.0001*** | 1.050[1.046-1.053];<0.0001*** |
| <50 | 1.0[Reference] | 1.0[Reference] | 1.0[Reference] | 1.0[Reference] |
| [50-60] | 0.53[0.47-0.58];<0.0001*** | 0.32[0.29-0.35];<0.0001*** | 0.77[0.69-0.86];<0.0001*** | 0.24[0.21-0.28];<0.0001*** |
| [60-70] | 0.69[0.63-0.75];<0.0001*** | 0.62[0.57-0.67];<0.0001*** | 0.56[0.49-0.64];<0.0001*** | 1.26[1.14-1.40];<0.0001*** |
| [70-80] | 1.78[1.63-1.94];<0.0001*** | 2.00[1.86-2.15];<0.0001*** | 4.48[4.06-4.95];<0.0001*** | 4.68[4.26-5.15];<0.0001*** |
| >80 | 3.42[3.13-3.73];<0.0001*** | 5.12[4.77-5.49];<0.0001*** | 0.99[0.85-1.16];0.9328 | 1.28[1.12-1.46];0.0003*** |
| ***Past comorbidities*** |  |  |  |  |
| Charlson standard comorbidity index | 1.35[1.33-1.38];<0.0001*** | 1.45[1.43-1.47];<0.0001*** | 1.28[1.24-1.32];<0.0001*** | 1.40[1.36-1.43];<0.0001*** |
| Diabetes with chronic complication | 2.11[1.61-2.77];<0.0001*** | 2.00[1.57-2.54];<0.0001*** | 2.56[1.90-3.45];<0.0001*** | 2.30[1.70-3.12];<0.0001*** |
| Diabetes without chronic complication | 1.23[0.94-1.61];0.1279 | 1.48[1.19-1.83];0.0003*** | 1.39[1.04-1.86];0.0251* | 1.63[1.26-2.10];0.0002*** |
| Gout | 2.17[1.82-2.59];<0.0001*** | 2.52[2.18-2.91];<0.0001*** | 0.91[0.63-1.31];0.6072 | 2.33[1.86-2.92];<0.0001*** |
| Hyperlipidaemia | 1.07[0.85-1.35];0.5799 | 0.85[0.68-1.06];0.1561 | 0.67[0.49-0.92];0.0124* | 0.42[0.28-0.61];<0.0001*** |
| Hypertension | 1.65[1.52-1.79];<0.0001*** | 1.91[1.78-2.05];<0.0001*** | 0.62[0.54-0.71];<0.0001*** | 1.53[1.38-1.69];<0.0001*** |
| Hypoglycemia | 2.97[2.25-3.91];<0.0001*** | 2.53[1.96-3.27];<0.0001*** | 0.54[0.14-2.18];0.3895 | 0.99[0.37-2.63];0.9791 |
| Ischemic heart disease | 1.91[1.69-2.14];<0.0001*** | 1.75[1.58-1.95];<0.0001*** | 3.34[2.99-3.74];<0.0001*** | 2.29[2.03-2.58];<0.0001*** |
| Liver diseases | 0.63[0.46-0.88];0.0068** | 0.84[0.65-1.07];0.1594 | 0.37[0.24-0.58];<0.0001*** | 0.58[0.41-0.82];0.0020** |
| Peripheral vascular disease | 3.64[2.79-4.75];<0.0001*** | 3.47[2.74-4.40];<0.0001*** | 2.33[1.42-3.81];0.0008*** | 2.51[1.60-3.94];0.0001*** |
| Renal diseases | 3.38[2.82-4.04];<0.0001*** | 2.58[2.17-3.07];<0.0001*** | 0.98[0.49-1.97];0.9635 | 1.36[0.77-2.40];0.2899 |
| Stroke/transient ischemic attack | 1.85[1.56-2.19];<0.0001*** | 2.17[1.89-2.49];<0.0001*** | 1.30[0.98-1.72];0.0639 | 1.85[1.47-2.32];<0.0001*** |
| Atrial fibrillation | 1.92[1.54-2.40];<0.0001*** | 4.46[3.89-5.10];<0.0001*** | 1.49[1.08-2.07];0.0158* | 3.91[3.19-4.79];<0.0001*** |
| VT/VF/aborted SCD | 1.49[0.56-3.98];0.4244 | 2.33[1.16-4.65];0.0171* | 1.38[0.44-4.27];0.5807 | 1.70[0.64-4.54];0.2878 |
| Anaemia | 1.97[1.69-2.29];<0.0001*** | 2.34[2.07-2.65];<0.0001*** | 1.77[1.37-2.28];<0.0001*** | 1.87[1.47-2.37];<0.0001*** |
| Overweight | 0.45[0.22-0.89];0.0229* | 0.80[0.51-1.26];0.3449 | 0.23[0.10-0.51];0.0003*** | 1.22[0.87-1.71];0.2488 |
| Cancer | 1.15[0.92-1.44];0.2209 | 1.58[1.33-1.87];<0.0001*** | 0.41[0.24-0.70];0.0010** | 1.16[0.85-1.57];0.3436 |
| ***Medications*** |  |  |  |  |
| SGLT2I v.s. DPP4I | 0.56[0.51-0.61];<0.0001*** | 0.39[0.36-0.42];<0.0001*** | 0.60[0.54-0.66];<0.0001*** | 0.52[0.48-0.58];<0.0001*** |
| SGLT2I frequency | 1.00[1.00-1.01];0.2400 | 1.01[1.00-1.01];0.0654 | 1.00[1.00-1.01];0.2400 | 1.01[1.00-1.01];0.0654 |
| DPP4I frequency | 1.00[0.99-1.00];0.1800 | 1.00[1.00-1.01];0.3181 | 0.96[0.95-0.97];<0.0001*** | 0.97[0.96-0.98];<0.0001*** |
| SGLT2I duration, days | 1.000[0.999-1.000];<0.0001*** | 0.999[0.999-1.000];<0.0001*** | 1.000[0.999-1.000];<0.0001*** | 0.999[0.999-1.000];<0.0001*** |
| DPP4I duration, days | 1.000[1.000-1.000];<0.0001*** | 1.000[1.000-1.000];<0.0001*** | 0.997[0.997-0.998];<0.0001*** | 0.999[0.999-1.000];<0.0001*** |
| Metformin | 0.40[0.36-0.44];<0.0001*** | 0.37[0.35-0.41];<0.0001*** | 0.83[0.70-0.99];0.0366* | 0.43[0.38-0.49];<0.0001*** |
| Sulphonylurea | 1.07[0.97-1.17];0.1581 | 1.03[0.95-1.12];0.4325 | 0.54[0.49-0.59];<0.0001*** | 0.44[0.40-0.48];<0.0001*** |
| Insulin | 4.78[4.32-5.27];<0.0001*** | 4.16[3.84-4.52];<0.0001*** | 2.18[1.96-2.41];<0.0001*** | 6.21[5.43-7.09];<0.0001*** |
| Acarbose | 0.89[0.68-1.15];0.3580 | 0.74[0.58-0.94];0.0142* | 2.07[1.73-2.47];<0.0001*** | 0.51[0.37-0.71];<0.0001*** |
| Thiazolidinedione | 0.39[0.34-0.44];<0.0001*** | 0.44[0.40-0.50];<0.0001*** | 0.26[0.22-0.31];<0.0001*** | 0.41[0.36-0.47];<0.0001*** |
| Glucagon-like peptide-1 receptor agonists | 0.42[0.30-0.59];<0.0001*** | 0.37[0.27-0.51];<0.0001*** | 0.37[0.27-0.50];<0.0001*** | 1.24[1.05-1.47];0.0113* |
| Statins and fibrates | 0.46[0.42-0.50];<0.0001*** | 0.37[0.34-0.40];<0.0001*** | 0.58[0.53-0.64];<0.0001*** | 0.47[0.43-0.52];<0.0001*** |
| ***Complete blood counts*** |  |  |  |  |
| Haemoglobin, g/dL | 0.80[0.79-0.82];<0.0001*** | 0.77[0.76-0.79];<0.0001*** | 1.05[1.01-1.09];0.0086** | 0.98[0.94-1.02];0.2501 |
| Mean corpuscular volume, fL | 1.02[1.01-1.03];<0.0001*** | 1.02[1.01-1.02];<0.0001*** | 1.10[1.09-1.11];<0.0001*** | 1.03[1.02-1.04];<0.0001*** |
| Eosinophil, x10^9/L | 1.17[1.04-1.31];0.0068** | 1.20[1.08-1.33];0.0006*** | 0.12[0.07-0.21];<0.0001*** | 0.32[0.21-0.50];<0.0001*** |
| Lymphocyte, x10^9/L | 0.70[0.65-0.75];<0.0001*** | 0.65[0.61-0.69];<0.0001*** | 0.96[0.88-1.06];0.4724 | 1.11[1.05-1.16];0.0001*** |
| Neutrophil, x10^9/L | 1.06[1.05-1.07];<0.0001*** | 1.05[1.04-1.06];<0.0001*** | 1.06[1.03-1.08];<0.0001*** | 1.13[1.12-1.15];<0.0001*** |
| White cell count, x10^9/L | 1.02[1.02-1.03];<0.0001*** | 1.02[1.02-1.03];<0.0001*** | 1.04[1.01-1.06];0.0057** | 1.12[1.11-1.14];<0.0001*** |
| Mean cell haemoglobin, pg | 1.06[1.04-1.08];<0.0001*** | 1.04[1.02-1.05];<0.0001*** | 1.25[1.21-1.29];<0.0001*** | 1.10[1.07-1.13];<0.0001*** |
| Platelet, x10^9/L | 0.999[0.998-1.000];0.0047** | 0.997[0.997-0.998];<0.0001*** | 0.998[0.997-0.999];0.0024** | 1.00[0.99-1.00];<0.0001*** |
| Red cell count, x10^12/L | 0.51[0.47-0.54];<0.0001*** | 0.47[0.44-0.50];<0.0001*** | 0.75[0.67-0.83];<0.0001*** | 0.78[0.70-0.86];<0.0001*** |
| ***Liver and renal functions*** |  |  |  |  |
| Potassium, mmol/L | 1.06[0.97-1.15];0.2222 | 1.08[1.00-1.16];0.0547 | 0.98[0.89-1.08];0.6892 | 0.66[0.59-0.73];<0.0001*** |
| Albumin, g/L | 0.89[0.88-0.90];<0.0001*** | 0.88[0.88-0.89];<0.0001*** | 1.05[1.03-1.07];<0.0001*** | 0.91[0.89-0.92];<0.0001*** |
| Sodium, mmol/L | 0.96[0.95-0.97];<0.0001*** | 0.98[0.96-0.99];0.0001*** | 1.22[1.20-1.24];<0.0001*** | 1.13[1.11-1.15];<0.0001*** |
| Urea, mmol/L | 1.08[1.08-1.09];<0.0001*** | 1.08[1.08-1.09];<0.0001*** | 1.07[1.06-1.08];<0.0001*** | 1.08[1.07-1.09];<0.0001*** |
| Protein, g/L | 0.95[0.94-0.96];<0.0001*** | 0.96[0.95-0.96];<0.0001*** | 0.96[0.95-0.98];<0.0001*** | 0.99[0.97-1.00];0.0393* |
| Creatinine, umol/L | 1.002[1.002-1.003];<0.0001*** | 1.002[1.002-1.002];<0.0001*** | 1.004[1.004-1.004];<0.0001*** | 1.004[1.004-1.005];<0.0001*** |
| Alkaline phosphatase, U/L | 1.004[1.003-1.004];<0.0001*** | 1.003[1.002-1.004];<0.0001*** | 1.00[0.99-1.00];0.0040** | 1.007[1.006-1.008];<0.0001*** |
| Aspartate transaminase, U/L | 1.001[1.001-1.002];<0.0001*** | 1.000[0.999-1.001];0.7658 | 0.96[0.95-0.97];<0.0001*** | 0.999[0.995-1.003];0.6592 |
| Alanine transaminase, U/L | 0.988[0.985-0.991];<0.0001*** | 0.99[0.98-0.99];<0.0001*** | 0.97[0.96-0.97];<0.0001*** | 0.98[0.98-0.99];<0.0001*** |
| Bilirubin, umol/L | 0.97[0.96-0.98];<0.0001*** | 0.99[0.98-1.00];0.0104* | 0.93[0.91-0.94];<0.0001*** | 1.00[0.99-1.01];0.5222 |
| ***Lipid and glucose profiles*** |  |  |  |  |
| Triglyceride, mmol/L | 1.03[1.01-1.05];0.0141* | 0.97[0.94-1.00];0.0629 | 1.02[0.99-1.05];0.1309 | 1.00[0.96-1.03];0.9458 |
| Low-density lipoprotein, mmol/L | 1.11[1.05-1.17];0.0001*** | 0.87[0.83-0.92];<0.0001*** | 1.07[1.00-1.15];0.0593 | 1.07[1.00-1.15];0.0520 |
| High-density lipoprotein, mmol/L | 0.63[0.54-0.73];<0.0001*** | 0.86[0.76-0.97];0.0144* | 0.31[0.26-0.38];<0.0001*** | 0.67[0.56-0.79];<0.0001*** |
| Total cholesterol, mmol/L | 1.07[1.02-1.11];0.0024** | 0.89[0.85-0.92];<0.0001*** | 0.97[0.92-1.03];0.3232 | 1.03[0.97-1.08];0.3681 |
| Glucose, mmol/L | 1.02[1.01-1.03];<0.0001*** | 1.01[1.00-1.02];0.2111 | 0.99[0.98-1.00];0.0781 | 1.01[1.00-1.02];0.1695 |

**Supplementary Table 3. Univariable Cox regression to identify significant predictors of cardiovascular mortality and all-cause mortality before and after 1:1 matching.**

* for p≤ 0.05, ** for p ≤ 0.01, *** for p ≤ 0.001; HR: hazard ratio; CI: confidence interval; SD: standard deviation; SCD: sudden cardiac death; VF: ventricular fibrillation; VT: ventricular tachycardia; SGLT2I: sodium glucose cotransporter-2 inhibitor; DPP4I: dipeptidyl peptidase-4 inhibitor; CV: coefficient of variation.

| **Characteristics** | **Before matching** |  | **After matching** |  |
| --- | --- | --- | --- | --- |
|  | **All-cause mortality**  **HR [95% CI];P value** | **Cardiovascular mortality**  **HR [95% CI];P value** | **All-cause mortality**  **HR [95% CI];P value** | **Cardiovascular mortality**  **HR [95% CI];P value** |
| ***Demographics*** |  |  |  |  |
| Male gender | 0.98[0.94-1.04];0.5475 | 1.15[1.05-1.26];0.0035** | 0.91[0.84-0.98];0.0143* | 0.96[0.81-1.14];0.6465 |
| Female gender | 1.0[Reference] | 1.0[Reference] | 1.0[Reference] | 1.0[Reference] |
| Baseline age, years | 1.09[1.09-1.10];<0.0001*** | 1.13[1.13-1.14];<0.0001*** | 1.049[1.046-1.052];<0.0001*** | 1.16[1.15-1.17];<0.0001*** |
| <50 | 1.0[Reference] | 1.0[Reference] | 1.0[Reference] | 1.0[Reference] |
| [50-60] | 0.26[0.24-0.28];<0.0001*** | 0.13[0.10-0.16];<0.0001*** | 0.56[0.51-0.61];<0.0001*** | 0.04[0.02-0.07];<0.0001*** |
| [60-70] | 0.54[0.51-0.58];<0.0001*** | 0.34[0.30-0.39];<0.0001*** | 0.31[0.27-0.35];<0.0001*** | 0.41[0.32-0.53];<0.0001*** |
| [70-80] | 1.96[1.86-2.07];<0.0001*** | 1.83[1.66-2.03];<0.0001*** | 3.51[3.24-3.80];<0.0001*** | 2.63[2.17-3.18];<0.0001*** |
| >80 | 6.80[6.46-7.15];<0.0001*** | 11.69[10.68-12.81];<0.0001*** | 2.60[2.38-2.84];<0.0001*** | 11.93[10.04-14.16];<0.0001*** |
| ***Past comorbidities*** |  |  |  |  |
| Charlson standard comorbidity index | 1.54[1.53-1.56];<0.0001*** | 1.61[1.58-1.63];<0.0001*** | 1.42[1.39-1.45];<0.0001*** | 1.79[1.74-1.85];<0.0001*** |
| Diabetes with chronic complication | 2.07[1.73-2.48];<0.0001*** | 1.56[1.07-2.26];0.0197* | 1.67[1.26-2.23];0.0004*** | 2.83[1.72-4.65];<0.0001*** |
| Diabetes without chronic complication | 1.53[1.31-1.80];<0.0001*** | 1.78[1.36-2.33];<0.0001*** | 1.81[1.48-2.21];<0.0001*** | 5.16[3.89-6.85];<0.0001*** |
| Gout | 2.30[2.06-2.57];<0.0001*** | 2.93[2.44-3.53];<0.0001*** | 1.65[1.33-2.05];<0.0001*** | 2.20[1.45-3.35];0.0002*** |
| Hyperlipidaemia | 0.83[0.70-0.99];0.0385* | 0.89[0.65-1.20];0.4308 | 0.54[0.41-0.71];<0.0001*** | 0.78[0.47-1.31];0.3548 |
| Hypertension | 1.92[1.82-2.02];<0.0001*** | 2.15[1.95-2.36];<0.0001*** | 1.47[1.35-1.59];<0.0001*** | 3.33[2.81-3.94];<0.0001*** |
| Hypoglycemia | 3.42[2.89-4.05];<0.0001*** | 3.89[2.91-5.19];<0.0001*** | 3.23[2.06-5.07];<0.0001*** | 6.12[2.91-12.91];<0.0001*** |
| Ischemic heart disease | 1.21[1.11-1.33];<0.0001*** | 1.17[0.99-1.39];0.0653 | 1.09[0.96-1.23];0.1931 | 1.97[1.57-2.47];<0.0001*** |
| Liver diseases | 1.04[0.88-1.24];0.6093 | 0.85[0.60-1.19];0.3375 | 0.73[0.57-0.95];0.0171* | 0.73[0.41-1.29];0.2749 |
| Peripheral vascular disease | 3.75[3.15-4.46];<0.0001*** | 3.78[2.77-5.17];<0.0001*** | 4.36[3.29-5.79];<0.0001*** | 3.20[1.52-6.74];0.0022** |
| Renal diseases | 4.32[3.89-4.81];<0.0001*** | 4.02[3.29-4.91];<0.0001*** | 2.13[1.47-3.09];0.0001*** | 6.66[4.11-10.80];<0.0001*** |
| Stroke/transient ischemic attack | 2.38[2.15-2.63];<0.0001*** | 3.03[2.57-3.57];<0.0001*** | 2.20[1.86-2.62];<0.0001*** | 7.15[5.64-9.07];<0.0001*** |
| Atrial fibrillation | 2.65[2.34-3.01];<0.0001*** | 3.37[2.75-4.14];<0.0001*** | 3.01[2.50-3.62];<0.0001*** | 6.85[5.12-9.17];<0.0001*** |
| VT/VF/SCD | 2.74[1.70-4.41];<0.0001*** | 2.12[0.79-5.66];0.1332 | 3.68[2.14-6.35];<0.0001*** | 5.79[2.16-15.48];0.0005*** |
| Anaemia | 3.06[2.81-3.33];<0.0001*** | 3.40[2.94-3.94];<0.0001*** | 2.16[1.80-2.59];<0.0001*** | 3.73[2.70-5.15];<0.0001*** |
| Overweight | 0.33[0.19-0.55];<0.0001*** | 0.08[0.01-0.55];0.0104* | 0.49[0.32-0.76];0.0013** | 0.00[0.00-Inf];0.9807 |
| Cancer | 2.29[2.06-2.55];<0.0001*** | 2.19[1.80-2.68];<0.0001*** | 2.03[1.67-2.47];<0.0001*** | 3.00[2.09-4.31];<0.0001*** |
| ***Medications*** |  |  |  |  |
| SGLT2I v.s. DPP4I | 0.16[0.15-0.17];<0.0001*** | 0.10[0.08-0.12];<0.0001*** | 0.23[0.21-0.26];<0.0001*** | 0.23[0.18-0.28];<0.0001*** |
| SGLT2I frequency | 1.02[1.01-1.02];<0.0001*** | 1.02[1.01-1.03];<0.0001*** | 1.02[1.01-1.02];<0.0001*** | 1.02[1.01-1.03];<0.0001*** |
| DPP4I frequency | 0.99[0.99-1.00];0.0018** | 1.00[0.99-1.00];0.2615 | 0.92[0.91-0.93];<0.0001*** | 1.00[0.99-1.01];0.8523 |
| SGLT2I duration, days | 1.000[1.000-1.000];0.0123* | 1.000[0.999-1.000];0.1366 | 1.000[1.000-1.000];0.0123* | 1.000[0.999-1.000];0.1366 |
| DPP4I duration, days | 1.000[1.000-1.000];<0.0001*** | 1.000[0.999-1.000];<0.0001*** | 0.998[0.998-0.998];<0.0001*** | 1.002[1.002-1.002];<0.0001*** |
| Metformin | 0.28[0.27-0.30];<0.0001*** | 0.28[0.25-0.31];<0.0001*** | 0.51[0.46-0.58];<0.0001*** | 0.26[0.21-0.32];<0.0001*** |
| Sulphonylurea | 1.06[1.00-1.13];0.0464* | 1.29[1.15-1.45];<0.0001*** | 0.36[0.33-0.39];<0.0001*** | 0.74[0.62-0.89];0.0012** |
| Insulin | 5.11[4.79-5.46];<0.0001*** | 7.85[6.83-9.03];<0.0001*** | 9.85[8.65-11.23];<0.0001*** | 6.71[5.23-8.61];<0.0001*** |
| Acarbose | 0.98[0.83-1.15];0.7939 | 0.95[0.71-1.28];0.7327 | 1.08[0.90-1.30];0.4066 | 2.86[2.17-3.77];<0.0001*** |
| Thiazolidinedione | 0.38[0.35-0.41];<0.0001*** | 0.27[0.23-0.33];<0.0001*** | 1.53[1.41-1.65];<0.0001*** | 0.17[0.12-0.24];<0.0001*** |
| Glucagon-like peptide-1 receptor agonists | 0.11[0.07-0.16];<0.0001*** | 0.05[0.02-0.16];<0.0001*** | 0.31[0.24-0.40];<0.0001*** | 1.15[0.85-1.58];0.3667 |
| Statins and fibrates | 0.30[0.29-0.32];<0.0001*** | 0.25[0.22-0.28];<0.0001*** | 0.46[0.42-0.49];<0.0001*** | 0.51[0.43-0.60];<0.0001*** |
| ***Complete blood counts*** |  |  |  |  |
| Haemoglobin, g/dL | 0.73[0.72-0.74];<0.0001*** | 0.72[0.70-0.74];<0.0001*** | 0.84[0.82-0.86];<0.0001*** | 0.77[0.73-0.81];<0.0001*** |
| Mean corpuscular volume, fL | 1.02[1.02-1.03];<0.0001*** | 1.02[1.01-1.03];<0.0001*** | 1.08[1.07-1.09];<0.0001*** | 1.08[1.07-1.10];<0.0001*** |
| Eosinophil, x10^9/L | 1.06[0.94-1.18];0.3601 | 0.89[0.68-1.18];0.4246 | 0.28[0.20-0.40];<0.0001*** | 0.07[0.03-0.18];<0.0001*** |
| Lymphocyte, x10^9/L | 0.53[0.50-0.56];<0.0001*** | 0.47[0.43-0.51];<0.0001*** | 0.97[0.90-1.04];0.3958 | 0.44[0.36-0.54];<0.0001*** |
| Neutrophil, x10^9/L | 1.05[1.05-1.06];<0.0001*** | 1.07[1.05-1.08];<0.0001*** | 1.09[1.08-1.11];<0.0001*** | 1.11[1.08-1.13];<0.0001*** |
| White cell count, x10^9/L | 1.02[1.01-1.02];<0.0001*** | 1.02[1.01-1.03];<0.0001*** | 1.05[1.04-1.07];<0.0001*** | 0.95[0.91-1.00];0.0674 |
| Mean cell haemoglobin, pg | 1.06[1.05-1.07];<0.0001*** | 1.04[1.02-1.06];0.0001*** | 1.19[1.16-1.21];<0.0001*** | 1.22[1.17-1.27];<0.0001*** |
| Platelet, x10^9/L | 0.997[0.997-0.998];<0.0001*** | 0.997[0.996-0.998];<0.0001*** | 1.003[1.002-1.004];<0.0001*** | 0.990[0.988-0.992];<0.0001*** |
| Red cell count, x10^12/L | 0.38[0.37-0.40];<0.0001*** | 0.38[0.35-0.41];<0.0001*** | 0.44[0.40-0.47];<0.0001*** | 0.35[0.30-0.41];<0.0001*** |
| ***Liver and renal functions*** |  |  |  |  |
| Potassium, mmol/L | 1.14[1.07-1.20];<0.0001*** | 1.19[1.07-1.32];0.0010** | 0.71[0.65-0.78];<0.0001*** | 1.20[0.99-1.44];0.0582 |
| Albumin, g/L | 0.859[0.855-0.864];<0.0001*** | 0.85[0.84-0.86];<0.0001*** | 0.93[0.92-0.94];<0.0001*** | 0.84[0.82-0.86];<0.0001*** |
| Sodium, mmol/L | 0.95[0.94-0.96];<0.0001*** | 0.95[0.93-0.96];<0.0001*** | 1.11[1.09-1.13];<0.0001*** | 0.99[0.96-1.03];0.7573 |
| Urea, mmol/L | 1.089[1.085-1.092];<0.0001*** | 1.08[1.08-1.09];<0.0001*** | 1.07[1.07-1.08];<0.0001*** | 1.07[1.06-1.09];<0.0001*** |
| Protein, g/L | 0.95[0.94-0.95];<0.0001*** | 0.94[0.93-0.95];<0.0001*** | 1.03[1.02-1.04];<0.0001*** | 1.01[0.98-1.03];0.6243 |
| Creatinine, umol/L | 1.003[1.002-1.003];<0.0001*** | 1.002[1.002-1.003];<0.0001*** | 1.004[1.004-1.004];<0.0001*** | 1.004[1.003-1.005];<0.0001*** |
| Alkaline phosphatase, U/L | 1.00[1.00-1.01];<0.0001*** | 1.00[1.00-1.01];<0.0001*** | 1.006[1.005-1.007];<0.0001*** | 1.001[0.996-1.005];0.7958 |
| Aspartate transaminase, U/L | 1.000[1.000-1.001];0.4442 | 1.000[0.998-1.002];0.9622 | 1.00[1.00-1.01];0.0001*** | 0.97[0.95-0.99];0.0003*** |
| Alanine transaminase, U/L | 0.99[0.98-0.99];<0.0001*** | 0.97[0.97-0.98];<0.0001*** | 0.99[0.98-0.99];<0.0001*** | 0.92[0.91-0.94];<0.0001*** |
| Bilirubin, umol/L | 0.99[0.98-0.99];0.0001*** | 0.97[0.96-0.98];<0.0001*** | 0.99[0.98-1.00];0.0077** | 0.96[0.93-0.98];0.0002*** |
| ***Lipid and glucose profiles*** |  |  |  |  |
| Triglyceride, mmol/L | 0.94[0.92-0.96];<0.0001*** | 0.87[0.83-0.92];<0.0001*** | 0.68[0.64-0.73];<0.0001*** | 0.75[0.65-0.86];0.0001*** |
| Low-density lipoprotein, mmol/L | 0.95[0.92-0.99];0.0104* | 0.86[0.80-0.93];0.0001*** | 0.89[0.84-0.95];0.0006*** | 0.82[0.71-0.96];0.0151* |
| High-density lipoprotein, mmol/L | 1.14[1.05-1.25];0.0026** | 1.47[1.26-1.71];<0.0001*** | 2.17[1.94-2.43];<0.0001*** | 3.23[2.57-4.06];<0.0001*** |
| Total cholesterol, mmol/L | 0.95[0.92-0.98];0.0014** | 0.89[0.84-0.94];0.0001*** | 0.90[0.85-0.95];0.0001*** | 0.97[0.86-1.09];0.6100 |
| Glucose, mmol/L | 1.02[1.01-1.02];<0.0001*** | 1.02[1.01-1.03];0.0002*** | 0.98[0.97-0.99];0.0028** | 1.01[0.99-1.03];0.5219 |
|  |  |  |  |  |

**Supplementary Table 4. Sensitivity analysis 1: Multivariable Cox models with a one-year lag time.**

* for p≤ 0.05, ** for p ≤ 0.01, *** for p ≤ 0.001; SGLT2I: Sodium-glucose cotransporter-2 inhibitors; DPP4I: Dipeptidyl peptidase-4 inhibitors; HR: hazard ratio; CI: confidence interval.

| **Adverse outcomes** | **SGLT2I v.s. DPP4I**  **HR [95% CI];P value** |
| --- | --- |
| All-cause mortality | 0.25[0.23-0.29];<0.0001*** |
| Cardiovascular mortality | 0.26[0.20-0.34];<0.0001*** |
| Myocardial infarction | 0.46[0.40-0.52];<0.0001*** |
| Heart failure | 0.40[0.35-0.46];<0.0001*** |

**Supplementary Table 5. Sensitivity analysis 2: Competing risk analyses.**

* for p≤ 0.05, ** for p ≤ 0.01, *** for p ≤ 0.001; SGLT2I: Sodium-glucose cotransporter-2 inhibitors; DPP4I: Dipeptidyl peptidase-4 inhibitors; HR: hazard ratio; CI: confidence interval.

| **Models** | **Outcomes** | **SGLT2I v.s. DPP4I**  **HR [95% CI];P value** |
| --- | --- | --- |
| ***Cause-specific hazard models*** | |  |
|  | All-cause mortality | 0.24[0.21-0.27];<0.0001*** |
|  | Cardiovascular mortality | 0.30[0.22-0.40];<0.0001*** |
|  | Myocardial infarction | 0.49[0.42-0.57];<0.0001*** |
|  | Heart failure | 0.45[0.39-0.53];<0.0001*** |
| ***Subdistribution hazard models*** | |  |
|  | All-cause mortality | 0.21[0.19-0.24];<0.0001*** |
|  | Cardiovascular mortality | 0.25[0.19-0.32];<0.0001*** |
|  | Myocardial infarction | 0.61[0.55-0.69];<0.0001*** |
|  | Heart failure | 0.56[0.50-0.62];<0.0001*** |

**Supplementary Table 6. Sensitivity analysis 3: Different propensity score approaches.**

* for p≤ 0.05, ** for p ≤ 0.01, *** for p ≤ 0.001; SGLT2I: Sodium-glucose cotransporter-2 inhibitors; DPP4I: Dipeptidyl peptidase-4 inhibitors; HR: hazard ratio; CI: confidence interval; PS: propensity score; IPTW: inverse probability of treatment weighting, SIPTW: stable inverse probability of treatment weighting.

| **Outcomes** | **PS stratification**  **HR [95% CI];P value** | **PS with IPTW**  **HR [95% CI];P value** | **PS with SIPTW**  **HR [95% CI];P value** |
| --- | --- | --- | --- |
| All-cause mortality | 0.26[0.11-0.36];<0.0001*** | 0.24[0.18-0.34];<0.0001*** | 0.27[0.18-0.36];<0.0001*** |
| Cardiovascular mortality | 0.31[0.28-0.51];<0.0001*** | 0.35[0.24-0.45];<0.0001*** | 0.32[0.24-0.45];<0.0001*** |
| Myocardial infarction | 0.51[0.45-0.69];<0.0001*** | 0.54[0.42-0.65];<0.0001*** | 0.60[0.32-0.65];<0.0001*** |
| Heart failure | 0.59[0.51-0.67];<0.0001*** | 0.63[0.31-0.69];<0.0001*** | 0.62[0.30-0.71];<0.0001*** |
